# *Botrytis elliptica* Infection Induces *LhSorPALs* Expression in *Lilium*: Overexpression of *LhSorPAL1* and *LhSorPAL2* Enhances Disease Resistance via Phenylpropane Metabolite Accumulation

**DOI:** 10.3390/plants15121797

**Published:** 2026-06-11

**Authors:** Yu Zou, Lijun Tan, Xiaoliang Zhao, Zhenhao Zhang, Qing Duan, Shunzhao Sui, Jing Li, Daofeng Liu

**Affiliations:** 1 Chongqing Engineering Research Center for Floriculture, Key Laboratory of Agricultural Biosafety and Green Production of Upper Yangtze River (Ministry of Education), College of Horticulture and Landscape Architecture, Southwest University, Chongqing 400715, China; raining1220@163.com (Y.Z.); tlj18807265712@163.com (L.T.); dugyg214@163.com (X.Z.); 13588571371@163.com (Z.Z.); sszcq@126.com (S.S.); 2 Flower Research Institute, Yunnan Academy of Agricultural Sciences, Kunming 650205, China; dq@yaas.org.cn

**Keywords:** *Lilium*, *Botrytis elliptica*, phenylalanine ammonia-lyase (PAL), disease resistance, phenylpropane metabolism

## Abstract

Phenylalanine ammonia-lyase (PAL) is the rate-limiting enzyme in the phenylpropane metabolic pathway, which is crucial for plant disease resistance. However, the functional roles of specific *PAL* members in lily defense against gray mold (*Botrytis elliptica*) remain unclear. Using the resistant lily cultivar ‘Sorbonne’, metabolomics analysis revealed that phenylpropane metabolites were significantly induced upon pathogen infection. Combined second- and third-generation transcriptome sequencing identified eight *PAL* family members. Among them, *LhSorPAL1* and *LhSorPAL2* were strongly induced by *B. elliptica* and were selected for further analysis. Both recombinant proteins exhibited PAL enzymatic activity catalyzing cinnamic acid production from L-phenylalanine. Overexpression of *LhSorPAL1* or *LhSorPAL2* in lily via Agrobacterium-mediated transformation had no obvious effect on plant growth but significantly increased the accumulation of lignin, flavonoids, and total phenols upon pathogen challenge, leading to enhanced resistance to gray mold. Conversely, antisense expression of *LhSorPAL1* or *LhSorPAL2* reduced the accumulation of these metabolites. Promoter analysis revealed that both *LhSorPAL1pro* and *LhSorPAL2pro* contain methyl jasmonate (MeJA)-, abscisic acid (ABA)-, and transcription factor-binding cis-elements. Collectively, these results demonstrate that *LhSorPAL1* and *LhSorPAL2* positively regulate lily resistance to *B. elliptica* by promoting phenylpropane metabolism, providing candidate genes for molecular breeding.

## 1. Introduction

Lily (*Lilium* spp.) is a bulbous perennial herb [1]. Owing to its diverse morphology, vibrant flower colors, and strong fragrance, lily possesses exceptional ornamental value and holds a prominent position in the global floriculture market, ranking among the top five cut flowers worldwide [2]. However, lily production is severely threatened by gray mold caused by *B. elliptica*, one of the most destructive diseases affecting lilies, leading to substantial economic losses [3]. Therefore, understanding the molecular mechanisms underlying lily resistance to *B. elliptica* is of great importance for breeding resistant cultivars and reducing reliance on chemical fungicides.

Plants are constantly exposed to various biotic stresses, including pathogen attack, in their natural environment. To counteract these challenges, they have evolved multiple defense strategies [4,5]. The production of secondary metabolites plays a central role in these processes, as many aspects of plant defense against pathogen attack are mediated by these compounds [5,6]. Plant secondary metabolites are considered essential for plant adaptation and defense. These metabolites are synthesized via secondary metabolic pathways, among which the phenylpropanoid pathway is one of the most important. This pathway produces various defense-related compounds, including lignin and flavonoids, thereby enhancing plant disease resistance through physical barrier reinforcement or direct antimicrobial activity [7,8].

Phenylalanine ammonia-lyase (PAL) is a key rate-limiting enzyme in the phenylpropanoid pathway. It is an oligomeric enzyme typically composed of four subunits, with a molecular weight ranging from 300 to 340 kDa [9]. PAL catalyzes the deamination of L-phenylalanine to produce trans-cinnamic acid, the first step of the phenylpropanoid pathway, thereby laying the foundation for subsequent metabolic reactions [10]. The number of *PAL* genes varies greatly among plant species, and *PAL* is typically encoded by a multigene family whose members exhibit distinct expression patterns and functional characteristics [11]. For example, *Arabidopsis thaliana* contains 4 *PAL* members [12], *Camellia sinensis* has 7 [13], and cucumber has 15 members [14]. Functional divergence among *PAL* family members has been widely documented. In *Arabidopsis*, cold stress significantly upregulates *PAL1* and *PAL2*, whereas *PAL3* and *PAL4* show no marked response [15]. The *AmPAL* gene from *Astragalus* enhances salt, alkali, and drought tolerance in transgenic tobacco [16]. In potato, the *StPAL* gene may be involved in defense mechanisms against high-temperature and drought stress [17].

Furthermore, PAL is recognized as a key component of plant defense against a wide range of biotic stresses [7]. In tomato infected with root-knot nematodes, *SlPAL5*, *SlPAL8*, *SlPAL11*, and *SlPAL12* are significantly upregulated, while *SlPAL3*, *SlPAL4*, and *SlPAL6* are downregulated [18]. In rice, among the nine *OsPAL* genes, *OsPAL6* and *OsPAL8* are specifically expressed in stems and leaf sheaths; overexpression of *OsPAL8* enhances resistance of susceptible rice varieties to brown planthopper (BPH), whereas overexpression of *OsPAL6* reduces resistance [19]. Enhanced expression of *SlPAL2* in tomato increases resistance to bacterial canker [20], while *SlPAL3* may be associated with resistance to leaf spot [21]. In wheat, *AevPAL1* confers resistance to cereal cyst nematode by influencing the synthesis of salicylic acid (SA) and downstream secondary metabolites [22]. Heterologous expression of *ShPAL* in tobacco significantly reduces lesion area, indicating that *PAL* plays a vital role in plant defense [23].

Despite extensive studies on *PAL* genes in various plant species and their demonstrated roles in plant defense through multiple pathways, research on *PAL* in *Lilium* remains limited. Existing studies have largely treated PAL as a biochemical entity, such as an enzyme or a metabolic indicator, rather than investigating it at the gene level. Studies have found that PAL activity shows no significant correlation with disease resistance in lily leaves following *B. elliptica* infection [24]. In contrast, in ‘Sorbonne’ lily leaves infected with *LMoV*, PAL activity, as well as total phenolic and flavonoid concentrations, was significantly increased [25]. At the transcriptomic level, a recent multi-omics study identified *PAL* as a key gene involved in lily defense against cotton aphids (*Aphis gossypii*), but did not further characterize its function [26]. Specifically, how *PAL* genes participate in disease resistance by regulating the phenylpropanoid pathway has not yet been clearly elucidated, and the molecular mechanisms of *LhSorPALs* remain to be investigated.

In our preliminary experiments using the highly resistant lily cultivar ‘Sorbonne’, metabolomic analysis after *B. elliptica* infection revealed a significant increase in phenylpropanoid metabolites, suggesting their involvement in lily resistance to gray mold.

To further investigate whether *PAL* genes enhance lily resistance to gray mold and to elucidate their molecular mechanisms and regulatory pathways, we identified eight *PAL* genes from the lily transcriptome database and characterized their phylogenetic relationships, tissue-specific expression, and response patterns to pathogen infection and phytohormones. *LhSorPAL1* and *LhSorPAL2*, which were significantly induced by *B elliptica*, were selected for functional studies. We then performed recombinant protein enzyme activity assays, constructed overexpression and antisense expression vectors, and generated transgenic lilies via genetic transformation of embryogenic callus. Functional disease resistance assays were conducted on the transgenic lines, and the promoter activities of *LhSorPAL1* and *LhSorPAL2* were analyzed. This study aims to characterize the function and mechanism of *PAL* genes in lily, thereby advancing our understanding of the biosynthesis and regulatory mechanisms of the phenylpropanoid pathway and providing a valuable reference for future research on the roles of secondary metabolites in plant growth, development, and stress responses.

## 2. Results

### 2.1. Identification and Phylogenetic Analysis of the PAL Gene Family in Lily

**Figure 1:**
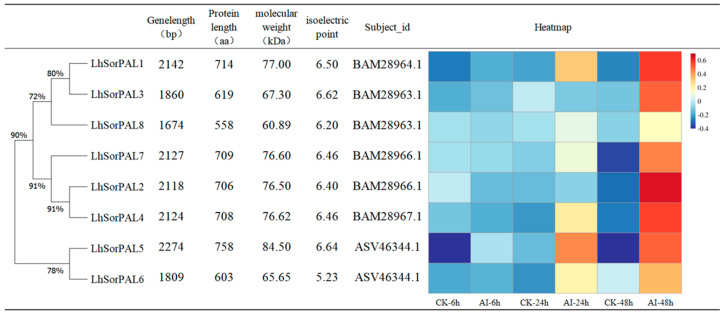
Phylogenetic relationships, protein sequence features, and expression profiles of *LhSorPALs* genes. Phylogenetic tree of the eight LhSorPAL proteins constructed using the neighbor-joining method. Amino acid length, molecular weight (MW), and theoretical isoelectric point (pI) of each LhSorPAL protein. Heatmap showing expression levels (FPKM, log2-transformed) of the eight *LhSorPALs* genes at 6, 24, and 48 hpi with *B. elliptica*. AI, after *B. elliptica* inoculation treatment; CK, mock-treated control.

Using second- and third-generation full-length transcriptomic data from ‘Sorbonne’ lily leaves infected by *B. elliptica* [27,28], we obtained the full-length sequences of eight *LhSorPALs* genes. Their physicochemical properties, including open reading frame (ORF) length, amino acid sequence length, protein molecular weight, and isoelectric point, were predicted and analyzed. The full-length sequences of the eight *LhSorPALs* genes ranged from 1674 bp to 2274 bp, with deduced amino acid lengths ranging from 558 to 758, protein molecular weights ranging from 60.89 kDa to 84.5 kDa, and isoelectric points ranging from 5.23 to 6.64. Phylogenetic analysis revealed that the eight *LhSorPALs* genes were broadly divided into three clades (Figure 1): *LhSorPAL1*, *LhSorPAL3*, and *LhSorPAL8* formed one cluster; *LhSorPAL2*, *LhSorPAL4*, and *LhSorPAL7* formed a second cluster; and *LhSorPAL5* and *LhSorPAL6* formed a separate cluster relatively distant from the others.

To assess the expression levels of the eight *PAL* genes following *B. elliptica* inoculation, a heatmap was generated based on fragments per kilobase of transcript per million mapped reads (FPKM) values at 6, 24, and 48 h post-inoculation (hpi), corresponding to the early, middle, and late stages of infection (Figure 1). Most *PAL* genes exhibited significant expression differences between the inoculated (AI) and the mock-treated control (CK), and these differences increased over time. With the exception of *LhSorPAL2* and *LhSorPAL3*, all other genes were significantly induced as early as 24 hpi. Notably, *LhSorPAL1* and *LhSorPAL2* showed the highest expression levels among all genes at 48 hpi, with highly significant differences between the inoculated and control samples.

### 2.2. Expression Profiles of LhSorPAL Genes in Different Tissues and in Response to B. elliptica Infection

#### 2.2.1. Tissue-Specific Expression of *LhSorPAL* Genes

**Figure 2:**
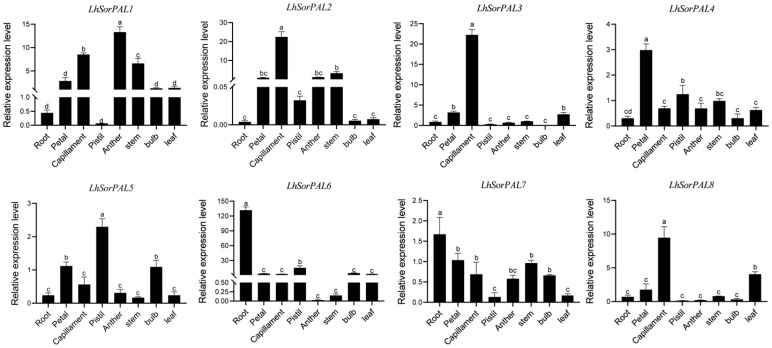
Tissue-specific expression of *LhSorPAL* genes in lily. Data are mean ± SD (n = 3). Different letters indicate significant differences (*p* < 0.05).

To investigate the potential functions of *LhSorPAL* genes in lily growth and development, we examined their expression profiles across various tissues using qRT-PCR (Figure 2). The eight *LhSorPAL* genes exhibited tissue-specific expression patterns: *LhSorPAL1* through *LhSorPAL5* and *LhSorPAL8* showed higher expression levels in floral tissues, whereas *LhSorPAL6* and *LhSorPAL7* were predominantly expressed in roots. These distinct expression patterns suggest that *LhSorPAL* genes may play diverse roles in lily growth and development.

#### 2.2.2. Expression of *LhSorPAL* Genes in Response to *B. elliptica* Infection

**Figure 3:**
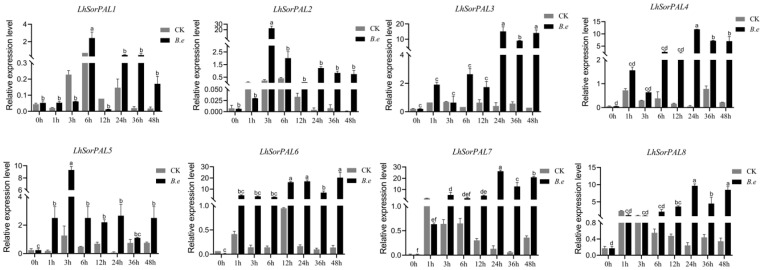
Time course of the level of the *LhSorPAL* genes in ‘Sorbonne’ after infection with *B. elliptica*. Expression levels of eight *LhSorPAL* genes were measured by qRT-PCR at 0, 1, 3, 6, 12, 24, 36, and 48 hpi. Each panel represents one gene. Data are presented as mean ± SD (n = 3). Different lowercase letters above the bars indicate significant differences among time points for each gene (*p* < 0.05).

To validate and extend the transcriptome-based expression data (Figure 1), we performed qRT-PCR to examine the expression profiles of the eight *LhSorPAL* genes in response to *B. elliptica* infection. Expression levels were compared between inoculated (AI) and mock-treated control (CK) tissues at different time points post-inoculation (Figure 3). The eight *LhSorPAL* genes were induced to varying degrees upon infection. *LhSorPAL2* and *LhSorPAL5* reached peak expression at 3 hpi, and *LhSorPAL1* peaked at 6 hpi, whereas *LhSorPAL3*, *LhSorPAL4*, *LhSorPAL6*, *LhSorPAL7* and *LhSorPAL8* were induced at later stages of infection. These results indicate that *LhSorPAL* genes are responsive to *B. elliptica* infection and may play a role in lily defense against this pathogen. To determine whether the responsiveness of *LhSorPAL* genes is specific to *B. elliptica* or represents a broader defense mechanism, we also examined their expression upon infection with *Botrytis cinerea*, a gray mold fungus with a broader host spectrum. All eight *LhSorPAL* genes were induced by *B. cinerea* as well, but with different temporal patterns, suggesting that *LhSorPALs* may play a broader role in lily defense against fungal pathogens (Figure S1).

### 2.3. Hormone Response Patterns of LhSorPAL Genes

**Figure 4:**
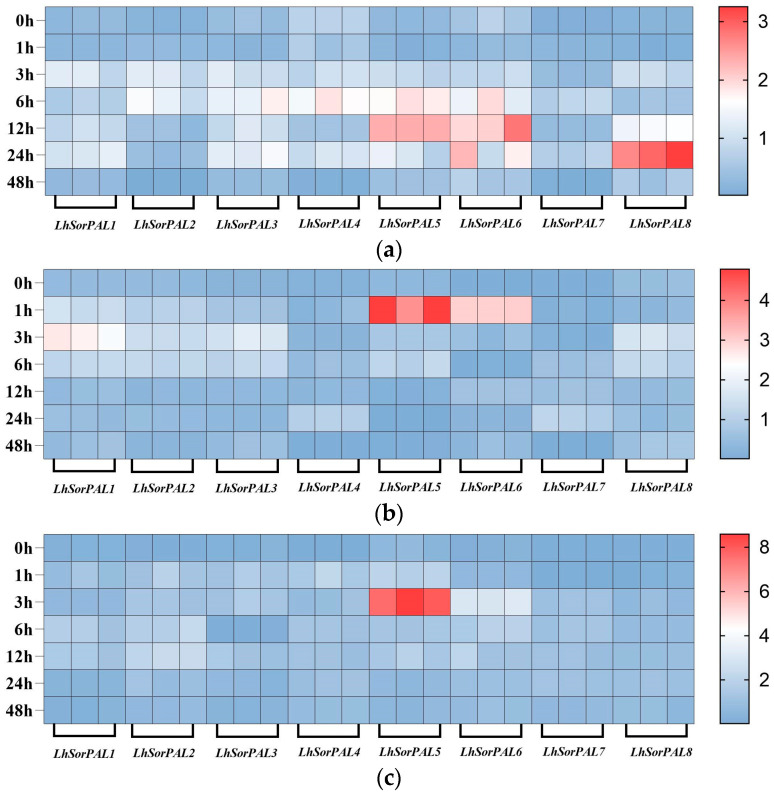
Expression patterns of *LhSorPAL* genes in response to hormone treatments. Heatmaps showing expression levels of eight *LhSorPAL* genes in response to (**a**) salicylic acid (SA), (**b**) methyl jasmonate (MeJA), and (**c**) abscisic acid (ABA) at 0, 1, 3, 6, 12, 24, and 48 h post-treatment. Red indicates upregulation; blue indicates downregulation. Each heatmap uses an independent color scale. Data represent mean values from three biological replicates.

To investigate the hormonal regulation of *LhSorPAL* genes, we examined their expression in response to salicylic acid (SA), methyl jasmonate (MeJA), and abscisic acid (ABA) at 0, 1, 3, 6, 12, 24, and 48 h post-treatment. The expression patterns of the eight *LhSorPAL* genes under each hormone treatment are presented as heatmaps (Figure 4). The eight *LhSorPAL* genes exhibited distinct temporal response patterns to these phytohormones. Some genes showed early and transient induction, whereas others displayed sustained or delayed responses. These results indicate that *LhSorPAL* genes are differentially regulated by defense-related and abiotic stress hormones, suggesting their potential involvement in diverse hormone-mediated signaling pathways.

### 2.4. Recombinant Expression and Enzymatic Characterization of LhSorPAL1 and LhSorPAL2

To investigate the function of *LhSorPAL* genes in lily, we selected representative members from different phylogenetic clades for cloning. *LhSorPAL1* from Clade I and *LhSorPAL2* from Clade II were successfully cloned (Figure S2) and used as representatives for functional characterization.

**Figure 5:**
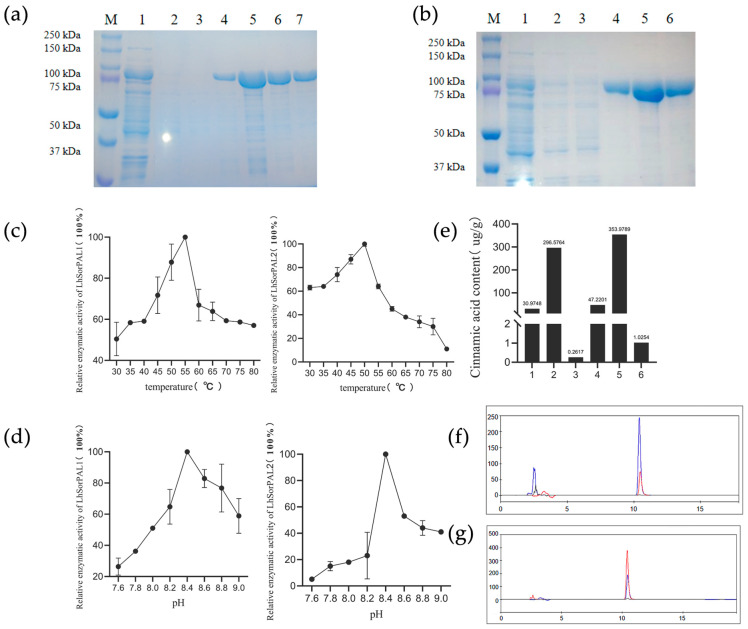
Analysis of recombinant protease activities of LhSorPAL1 and LhSorPAL2. (**a**) SDS-PAGE analysis of expressed and purification of recombinant pET32a(+)-LhSorPAL1. M: Protein molecular quality standard; 1: cell lysate; 2: flow through; 3: wash; 4–7: elution. (**b**) SDS-PAGE analysis of expressed and purification of recombinant pET32a(+)-LhSorPAL2. 1: cell lysate; 2: flow through; 3: wash; 4–6: elution. (**c**) Effect of pH on the enzymatic reactions of LhSorPAL1 and LhSorPAL2 in lilies. (**d**) Effect of Temperature on the Enzymatic Reactions of Lily LhSorPAL1 and LhSorPAL2. (**e**) HPLC detection of LhSorPAL1 and LhSorPAL2 enzymatic reaction products: 1: LhSorPAL1 reacted at 55 °C for 5 min; 2: LhSorPAL1 fully reacted at 55 °C; 4: LhSorPAL2 reacted at 50 °C for 5 min; 5: LhSorPAL2 fully reacted at 50 °C; 3, 6: blank control. (**f**) LhSorPAL1 high-performance liquid chromatography data: blue peak line: reaction sample; red peak line: cinnamic acid standard; black peak line: blank control. (**g**) LhSorPAL2 HPLC data: blue peak line: reaction sample; red peak line: cinnamic acid standard; black peak line: blank control.

To investigate the enzymatic properties of LhSorPAL1 and LhSorPAL2, we constructed prokaryotic expression vectors, expressed the recombinant proteins in *E. coli* BL21(DE3), and purified the enzymes. Both enzymes showed maximal activity at pH 8.8, with LhSorPAL1 exhibiting optimal activity at 55 °C and LhSorPAL2 at 50 °C. Activity assays confirmed that both recombinant proteins catalyze the deamination of L-phenylalanine to produce trans-cinnamic acid (Figure 5). These results demonstrate that both LhSorPAL1 and LhSorPAL2 encode functional PAL enzymes capable of initiating the phenylpropanoid pathway.

### 2.5. Overexpression of LhSorPAL1 and LhSorPAL2 in Lily Enhances Resistance to B. elliptica

**Figure 6:**
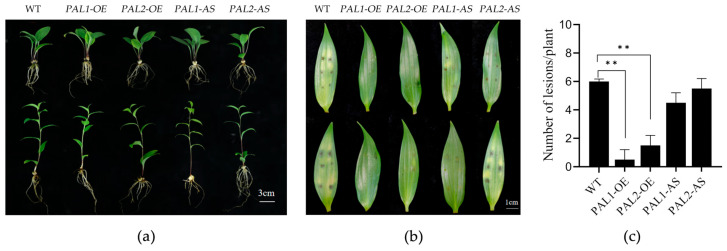
Analysis of fungal resistance of transgenic lilies. (**a**) Phenotypic observations of wild-type and transgenic lilies. (**b**) Disease symptoms of wild-type and transgenic lily leaves after *B. elliptica* inoculation. (**c**) Quantification of lesion areas. Data are presented as mean ± SD (n ≥ 3). Asterisks indicate statistically significant differences compared to the wild-type control (** *p* < 0.01). WT: wild type. OE: overexpression strain. AS: antisense expression strain. Due to the consistent phenotypes observed among independent transgenic lines, only one representative line per construct, exhibiting typical phenotype and good growth, is shown.

To investigate their roles in disease resistance, we constructed overexpression and antisense expression vectors for *LhSorPAL1* and *LhSorPAL2* and introduced them into lily via Agrobacterium-mediated transformation (Figure S3). The transgenic lines showed no obvious growth or morphological differences compared to wild-type plants (Figure 6). The transgenic lines and wild-type controls were inoculated with *B. elliptica*. Disease symptoms were assessed at 96 hpi. WT leaves developed numerous lesions and yellowing, whereas overexpression lines exhibited only mild lesions. In contrast, antisense expression lines showed more severe lesions (Figure 6). Consistent with these phenotypes, chlorophyll content was significantly higher in overexpression lines and significantly lower in antisense lines compared to wild-type controls after infection (Figure S4). These results indicate that overexpression of *LhSorPAL1* or *LhSorPAL2* confers enhanced resistance to *B. elliptica* in lily.

**Figure 7:**
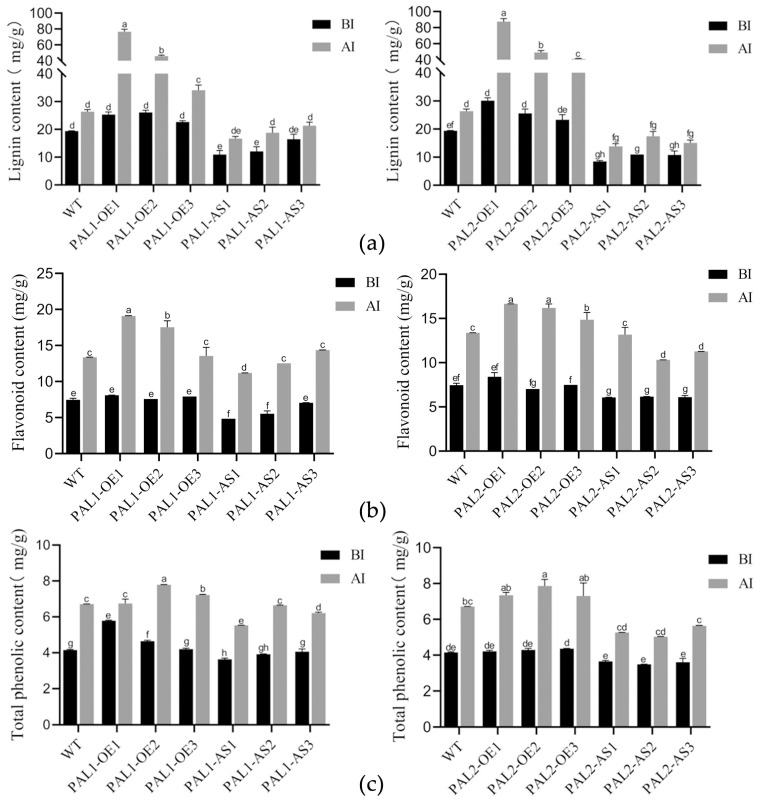
Accumulation of phenylpropanoid metabolites in wild-type and transgenic lily lines after *B. elliptica* infection. (**a**) Lignin content of *LhSorPAL1* and *LhSorPAL2* overexpression and antisense plants. (**b**) Flavonoid content of *LhSorPAL1* and *LhSorPAL2* overexpression and antisense plants. (**c**) Total phenol content of *LhSorPAL1* and *LhSorPAL2* overexpression and antisense plants. WT: wild type. OE: overexpression strain. AS: antisense expression strain. BI: before *B. elliptica* inoculation treatment. AI: after *B. elliptica* inoculation treatment. Three biological replicates were performed for each treatment. Different lowercase letters indicate statistically significant differences between groups (*p* < 0.05).

To understand how *LhSorPAL1* and *LhSorPAL2* affect disease resistance, we measured the accumulation of key phenylpropanoid metabolites—lignin, flavonoids, and total phenolics—in transgenic lines after *B. elliptica* infection. Overexpression lines accumulated significantly higher levels of these metabolites, whereas antisense lines showed reduced accumulation or no significant change (Figure 7). These results indicate that *LhSorPAL1* and *LhSorPAL2* positively regulate lily resistance to *B. elliptica* by promoting phenylpropanoid metabolism.

### 2.6. Promoter Analysis of LhSorPAL1 and LhSorPAL2

**Figure 8:**
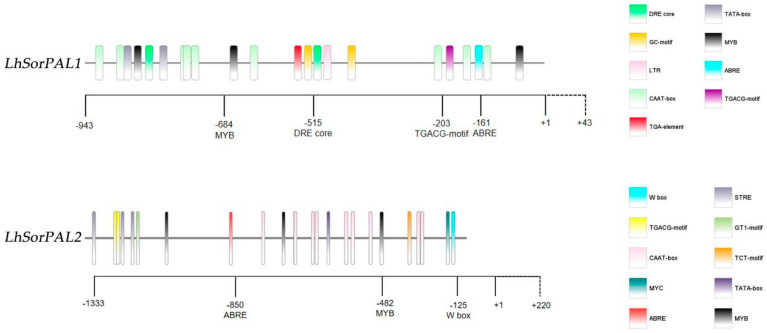
Analysis and segmentation of promoter sequences.

To gain insight into the transcriptional regulation of *LhSorPAL1* and *LhSorPAL2*, we cloned their promoter regions (Figure S5). Sequence analysis revealed that both promoters contain multiple cis-regulatory elements, including the MeJA-responsive TGACG-motif, the ABA-responsive ABRE, and W-boxes involved in SA responsiveness (Figure 8; Tables S1 and S2). Notably, this finding is consistent with the results in Section 2.3, which show that *LhSorPAL1* and *LhSorPAL2* are induced by SA, MeJA and ABA (Figure 4). Overall, this result provides potential transcriptional evidence for the hormonal regulation of these two genes, further supporting their role in lily resistance against *B. elliptica*.

## 3. Discussion

Lily is an important ornamental crop with substantial economic value. However, lily production is severely threatened by various diseases, particularly gray mold caused by *Botrytis* species. Prolonged use of chemical pesticides has led to the emergence of resistant pathogen strains [29]. Therefore, identifying disease-resistance genes through genetic engineering represents a promising strategy to enhance lily resistance.

Through second- and third-generation transcriptomic analyses, we identified the phenylalanine ammonia-lyase (*PAL*) gene family involved in the phenylpropanoid pathway associated with gray mold resistance in lily. *PALs* are typically encoded by multigene families, and the number of *PAL* genes varies considerably among plant species. In this study, we identified eight *PAL* genes in the ‘Sorbonne’ lily. By comparison, 14 *PAL* genes have been reported in potato [17], 11 in ginkgo [30], and seven in alfalfa [31]. Protein domain analysis revealed that all eight LhSorPAL proteins contain the conserved PAL-HAL domain. However, their expression patterns showed marked tissue specificity, which is consistent with observations in tea, where seven *CsPAL* genes also exhibited tissue-specific expression [13]. These results suggest that the divergent expression patterns of *PAL* family members may be closely associated with functional differentiation during plant development and stress responses.

The dynamic expression of plant *PAL* genes during pathogen infection is closely associated with their roles in disease resistance. In this study, infection by both *B. cinerea* and *B. elliptica* activated the expression of *LhSorPAL* genes in ‘Sorbonne’ lily. Upon *B. cinerea* infection, *LhSorPAL1*, *2*, *4*, *6*, *7*, and *8* were significantly upregulated as early as 1 hpi, followed by a rapid decline, exhibiting characteristics of an early defense response. This rapid induction pattern contrasts with that of the cucumber *CsPAL* gene during powdery mildew infection, which showed significant upregulation only at 16 hpi and peaked at 24 hpi [32], suggesting that different pathogen effectors may regulate *PAL* expression through distinct signaling pathways. In contrast, upon *B. elliptica* infection, *LhSorPAL* genes exhibited a sustained induction pattern.

Plant hormones play a crucial role in regulating plant growth, development, and adaptation to biotic and abiotic stresses [33]. Environmental factors and endogenous signals together constitute the regulatory network governing *PAL* gene expression. In this study, treatments with SA, MeJA, and ABA all induced differential expression of *LhSorPAL* genes, consistent with findings in walnut [34] and *Dendrobium* [35]. This indicates that *LhSorPAL* expression is integrated into multiple hormone signaling pathways. SA and JA are important defense signaling molecules that both contribute to plant resistance against pathogens. The fact that both SA and JA induced *LhSorPAL* expression is consistent with previous studies in maize [36], cucumber [37], and tomato [38], suggesting that in lily, SA and JA signaling pathways may not be strictly antagonistic but rather cooperate to activate defense responses against *B. elliptica*. ABA, a key hormone in abiotic stress responses such as drought and high salinity, also induced *LhSorPAL* expression in this study. This finding is consistent with the observation that elevated ABA levels in *Brassica napus* contribute to enhanced resistance against *Leptosphaeria maculans* [39]. As representative genes of the *LhSorPAL* family, *LhSorPAL1* and *LhSorPAL2* both contain the MeJA-responsive TGACG-motif and the ABA-responsive ABRE element in their promoters. Additionally, the promoter of *LhSorPAL2* contains a W-box element involved in SA responsiveness. These findings suggest that *LhSorPALs* may be directly regulated by these phytohormones at the transcriptional level.

In this study, we generated transgenic lily lines overexpressing or antisense-expressing *LhSorPAL1* and *LhSorPAL2*. No obvious phenotypic differences were observed between transgenic and wild-type plants under normal growth conditions. This may reflect a strategy in which *LhSorPAL1* and *LhSorPAL2* prioritize chemical defense over morphological changes. The role of *PAL* genes in disease resistance appears to be conserved across species. In pear, overexpression of *PbPAL* in *Arabidopsis* led to increased vessel wall thickness and higher lignin content [33]. In rice, *OsPAL* RNAi lines showed reduced lignin content and increased susceptibility to planthoppers compared to wild-type plants [19]. In our study, lily lines overexpressing *LhSorPAL1* or *LhSorPAL2* exhibited increased levels of lignin, flavonoids, and total phenolics after *B. elliptica* infection. Moreover, a correlation was observed between lesion expansion and lignin accumulation rate. Similar results have been reported in citrus, where the *CsPAL* gene family enhanced fruit resistance to *Penicillium* by increasing flavonoid and total phenolic content [40]. In contrast, antisense lines in our study showed relatively modest phenotypic changes. However, *PAL* silencing has been reported to have pronounced effects in other species. For example, suppression of *PAL1* in *Capsicum* led to a significant reduction in PAL activity and SA accumulation, thereby increasing susceptibility to *Xanthomonas campestris* [41].

## 4. Materials and Methods

### 4.1. Plant Materials

*Lilium* ‘Sorbonne’ bulbs were purchased from the Netherlands and grown in a greenhouse at Southwest University under controlled conditions (25 °C, 16 h light/8 h dark photoperiod, 20,000 Ix). Plants were cultivated in a peat soil:perlite:vermiculite mixture (1:1:1, *v*/*v*/*v*).

### 4.2. Strains and Vectors

*B. elliptica* (ACCC No. 36423) was purchased from the China Agricultural Microbial Strain Preservation and Management Centre (CAMSMC). *B. cinerea* was kindly provided by the School of Plant Protection, Southwest University, China. Both fungal strains were cultured on potato dextrose agar (PDA) medium (Hopebio, Qingdao, China) at 25 °C in the dark. The plant expression vector pVM01-GFP, which carries glyphosate and kanamycin resistance, was kindly provided by the Maize Experimental Group, College of Agronomy and Biotechnology, Southwest University. The pET-32a(+) vector was from the laboratory stock. pMD19-T vector was purchased from TaKaRa (Dalian, China). *Escherichia coli* DH5α and *E. coli* BL21(DE3) were purchased from TsingkeBiotech (Beijing, China). *Agrobacterium tumefaciens* strain EHA105 (rifampicin-resistant) was purchased from Weidi Biotech (Shanghai, China).

### 4.3. Identification and Bioinformatics Analysis of the LhSorPAL Gene Family

Full-length sequences of *PAL* genes were obtained from the second- and third-generation full-length transcriptome databases of ‘Sorbonne’ lily and translated into amino acid sequences. The resulting sequences were compared and analyzed using NCBI BLAST (https://blast.ncbi.nlm.nih.gov/Blast.cgi, accessed on 10 June 2024) to identify conserved domains. Sequences containing complete conserved domains were retained as candidate *LhSorPAL* genes. Phylogenetic tree analysis was performed using MEGA 6.0, and multiple amino acid sequence alignment was conducted using DNAMAN 8.0 software.

### 4.4. Characterisation of Induced Expression and Analysis of Tissue Expressivity of LhSorPALs Gene in Lily

For tissue-specific expression analysis, bulbs, scales, leaves, stems, roots, petals, anthers, and filaments were collected from ‘Sorbonne’ lily plants at full flowering stage. All samples were immediately frozen in liquid nitrogen and stored at −80 °C.

*B. elliptica* and *B. cinerea* were cultured on PDA medium at 25 °C for 7 days. Mycelial discs (1 cm in diameter) were prepared and inoculated onto mature leaves of ‘Sorbonne’ lily. BPlain agar discs were used as controls. Leaf samples were collected at 0, 1, 3, 6, 12, 24, and 48 hpi. For each time point, three leaves were randomly pooled as one biological replicate, and three independent replicates were performed. All samples were immediately frozen in liquid nitrogen and stored at −80 °C.

For hormone treatments, after selecting uniformly sized ‘Sorbonne’ lily tissue-cultured plantlets, they were pre-cultured in sterile water for 24 h. They were then transferred to solutions containing 100 μM MeJA, 200 μM SA, or 1 mM ABA for 30 min. After treatment, the plantlets were returned to sterile water for further incubation. Leaves were collected at 0, 1, 3, 6, 12, 24, and 48 h after treatment. Three biological replicates were performed for each treatment. Samples were flash-frozen in liquid nitrogen and stored at −80 °C.

Total RNA was extracted using TRIzol™ reagent (Thermo Fisher Scientific, Waltham, MA, USA) following the manufacturer‘s instructions. First-strand cDNA was synthesized using the All-in-One First-Strand Synthesis MasterMix (with dsDNase) reverse transcription kit (Yugong Biotech, Lianyungang, China). Gene-specific primers for the eight *LhSorPAL* genes were designed using Primer Premier 6.0 software based on transcriptome sequences (Table 1). qRT-PCR was performed using SsoFast EvaGreen Supermix (Bio-Rad, Hercules, CA, USA) under the following conditions: 95 °C for 30 s, followed by 40 cycles of 95 °C for 5 s and 58 °C for 5 s. The qRT-PCR system used was a CFX96 Touch Real-Time PCR Detection System (Bio-Rad, Hercules, CA, USA). Each reaction was performed with three technical replicates.

### 4.5. Cloning of LhSorPAL1 and LhSorPAL2 Genes

Gene-specific primers for *LhSorPAL1* and *LhSorPAL2* were designed using Primer Premier 6.0 based on the transcriptome sequences (Table 2). PCR amplification was performed using the above cDNA as a template on a Bio-Rad thermal cycler (Bio-Rad, Hercules, CA, USA). The thermal cycling conditions were as follows: 94 °C for 5 min; 32 cycles of 94 °C for 30 s, 50 °C for 30 s, and 72 °C for 2 min 30 s; and a final extension at 72 °C for 10 min. The PCR products were separated by electrophoresis on a 1% agarose gel, and the target fragments were excised and purified. The purified fragments were ligated into the pMD19-T vector and transformed into *E. coli* competent cells. Positive clones were identified by PCR and confirmed by Sanger sequencing (Tsingke Biotechnology Co., Chongqing, China). Sequence alignment was performed using MegAlign Pro 17.6 to obtain the final coding sequences.

### 4.6. Prokaryotic Expression and Recombinant Protein Analysis of LhSorPAL1 and LhSorPAL2

Primers containing homologous arms were designed based on the coding sequences of *LhSorPAL1* and *LhSorPAL2* (Table 2). The pET-32a(+) vector was digested with restriction enzymes and gel-purified. The target fragments were amplified by PCR and assembled into the linearized vector using homologous recombination. The recombinant plasmids were transformed into *E. coli* DH5α, and positive clones were confirmed by Sanger sequencing. The resulting plasmids were designated as pET32a(+)-*LhSorPAL1* and pET32a(+)-*LhSorPAL2*.

The recombinant plasmids pET32a(+)-*LhSorPAL1*, pET32a(+)-*LhSorPAL2*, and the empty pET32a(+) vector were transformed into *E. coli* strain BL21(DE3) for protein expression. Transformed cells were cultured at 37 °C with shaking at 180 rpm until the OD_600_ reached 0.6–0.8, as measured with a 722S visible spectrophotometer (Lengguang Technology, Shanghai, China). Protein expression was then induced with 0.02 mM IPTG (isopropyl-β-D-thiogalactopyranoside, Promega, Madison, WI, USA) at 28 °C with shaking at 180 rpm for 8 h. After induction, the bacterial cells were harvested by centrifugation at 12,000 rpm for 2 min. The supernatant was discarded, and the pellet was resuspended in PBS buffer (pH 7.4). The resuspended pellet was mixed with 4× SDS-PAGE loading buffer at a 3:1 ratio (*v*/*v*) in a sterile 1.5 mL tube, heated at 100 °C for 10 min, and centrifuged at 12,000 rpm for 2 min. The supernatant (10 μL) was subjected to SDS-PAGE electrophoresis. The recombinant protein was then purified using a His-tag protein purification kit (Beyotime Biotech, Shanghai, China) for subsequent activity assays.

### 4.7. Determination of Recombinant PAL Enzyme Activity

To determine the optimal pH and temperature for enzyme activity, the reaction mixture contained 900 μL of 0.1 M boric acid buffer (pH 7.4–9.0), 100 μL of 0.01 M L-phenylalanine substrate (dissolved in 0.1 M boric acid buffer, pH 8.8), and 100 μL of recombinant LhSorPAL1 or LhSorPAL2 protein. The reaction was incubated at 35 °C for 5 min, and absorbance at 290 nm (OD_290_) was measured using a microplate reader. For temperature optimization, the reaction was performed at temperatures ranging from 30 °C to 80 °C at optimal pH. The highest activity was defined as 100%, and relative activities were calculated accordingly. A control without enzyme was included. All experiments were performed in triplicate.

To analyze the reaction products, 100 μL of LhSorPAL1 or LhSorPAL2 enzyme solution was added to 100 μL of 0.01 M L-phenylalanine substrate (pre-incubated at 55 °C for LhSorPAL1 or 50 °C for LhSorPAL2 for 10 min), making a total reaction volume of 1 mL. The mixture was gently mixed and incubated at the respective temperature for 5 min and 12 min. A control without enzyme was included. An aliquot of the reaction mixture was sent to Chongqing Huakaifeichuang Biotechnology (Chongqing, China) for external analysis, and the remaining sample was analyzed by high-performance liquid chromatography (HPLC) using a Rigol L3000 system (Rigol, Beijing, China) equipped with a Sepax C18 reversed-phase column (250 mm × 4.6 mm, 5 μm).

### 4.8. Construction of Plant Overexpression and Antisense Expression Vectors for LhSorPAL1 and LhSorPAL2

For overexpression vector construction, *BamHI* was selected as the restriction site, and gene-specific primers were designed accordingly. For antisense expression vector construction, *BamHI* and *SacI* were selected as restriction sites, and the corresponding primers were designed (Table 3). Vector construction was performed following the method described in Section 4.6. The resulting vectors were designated as OE-pVM01-*LhSorPAL1*, OE-pVM01-*LhSorPAL2*, Anti-pVM01-*LhSorPAL1*, and Anti-pVM01-*LhSorPAL2*.

### 4.9. Genetic Transformation and Identification of Transgenic Lily Lines

The recombinant plasmids were introduced into *Agrobacterium tumefaciens* strain EHA105 using the freeze–thaw method according to the manufacturer’s instructions and confirmed by PCR. For genetic transformation, embryogenic calli derived from lily scales were incubated with the Agrobacterium suspension (OD_600_ = 0.6–0.8) on a low-speed shaker in the dark for 2–3 h. The calli were then transferred to co-culture medium and incubated in the dark for 3 days. After co-culture, the tissues were washed with 500 mg/L cefotaxime (Cef) solution and transferred to screening medium I for 15 days in the dark, followed by screening medium II under light conditions. Newly formed calli were subsequently transferred to screening medium III to induce bud differentiation, with subculturing every 20–30 days. After 60 days, the regenerated shoots were transferred to rooting medium, and putative transgenic plantlets were obtained. The compositions of all media are provided in Table 4.

Genomic DNA was extracted from the putative transgenic plantlets using the CTAB method, and PCR was performed to confirm the integration of the transgenes. Total RNA was extracted from PCR-positive plants using Trizol reagent, reverse-transcribed into cDNA, and subjected to qRT-PCR analysis as described in Section 4.4. Wild-type plants were used as controls to compare the expression levels of *LhSorPAL1* and *LhSorPAL2* in the transgenic lines.

### 4.10. Disease Resistance Assay of Transgenic Lily Lines Against B. elliptica

One-year-old tissue-cultured plantlets were taken out, washed with water to remove residual medium, and transplanted to the culture room. The substrate consisted of peat soil:perlite:vermiculite (1:1:1, *v*/*v*/*v*). Plants were grown at 25 °C under a 16 h light/8 h dark photoperiod with a light intensity of 20,000 lx. After one month, phenotypic observations were recorded, and growth parameters including plant height, leaf length, leaf width, root length, and bulb diameter, as well as the numbers of leaves and roots, were measured. Ten plantlets were randomly selected from each transgenic line and the wild-type control.

For disease resistance assays, healthy transgenic lines (both overexpression and antisense) were selected. Leaves were excised from the petiole, wrapped with sterile water-moistened cotton, and placed on cutting discs lined with filter paper. *B. elliptica* was cultured on PDA medium for 7 days, and mycelial discs (6 mm in diameter) were prepared using a hole puncher. The mycelial discs were placed upside down on the leaves. A small amount of sterile water was sprayed onto the leaves, and the cutting discs were sealed with plastic wrap. Inoculated leaves were incubated in the dark at 25 °C with 60% humidity for 96 h. Wild-type leaves inoculated with blank PDA discs served as controls. Three plants per line and three leaves per plant were used. Disease symptoms were photographed, and lesion areas were measured.

For physiological measurements, fresh leaf samples were collected. Half were used for chlorophyll content determination, and the other half were dried for measurement of lignin, flavonoids, and total phenolics. Chlorophyll content was determined using the acetone–ethanol extraction method [42]. Lignin content was determined using the acetyl bromide method [43]. Total flavonoid content was measured using the aluminum chloride colorimetric method [44]. Total phenolic content was measured using the Folin–Ciocalteu colorimetric method [45].

### 4.11. Promoter Element Analysis of the LhSorPAL1 and LhSorPAL2 Genes

The promoter fragments were cloned using a chromosome walking approach with the KX Genome Walking Kit (ZT601, Zoman Biotech, Beijing, China). Based on the validated known sequences, forward-specific primers were designed to amplify the unknown upstream regions (Table 5). The PCR products were separated by electrophoresis on a 1% agarose gel, and the target fragments were excised and purified. The purified fragments were ligated into the pMD19-T cloning vector and transformed into *E. coli* DH5α competent cells. Positive clones were screened and confirmed by Sanger sequencing. The cis-regulatory elements in the promoter sequences were predicted using the online software PlantCARE (http://bioinformatics.psb.ugent.be/webtools/plantcare/html/, accessed 5 December 2024).

### 4.12. Statistical Analysis

All data are presented as mean ± SD from three biological replicates. Student’s *t*-test was used for two-group comparisons, and one-way ANOVA with Tukey’s post hoc test was used for multiple comparisons. Differences were considered significant at *p* < 0.05. All statistical analyses were performed using GraphPad Prism 9.5.

## 5. Conclusions

In this study, we systematically characterized the *PAL* gene family in lily and functionally validated two members, *LhSorPAL1* and *LhSorPAL2*, in resistance against *B. elliptica*. Eight *PAL* genes were identified from transcriptomic data and classified into three phylogenetic clades. Among them, *LhSorPAL1* and *LhSorPAL2* were successfully cloned and shown to encode functional PAL enzymes. Overexpression of the *LhSorPAL1* and *LhSorPAL2* genes in lily significantly enhanced resistance to *B. elliptica*, whereas antisense expression compromised disease resistance. Mechanistically, *LhSorPAL1* and *LhSorPAL2* promoted the accumulation of lignin, flavonoids, and total phenolics, key metabolites of the phenylpropanoid pathway. Furthermore, both promoters contained MeJA- and ABA-responsive cis-elements, suggesting potential transcriptional regulation by these phytohormones. Together, these findings demonstrate that *LhSorPAL1* and *LhSorPAL2* positively regulate lily resistance to *B. elliptica* by promoting phenylpropane metabolism, providing candidate genes for molecular breeding of disease-resistant lily cultivars.

## Figures and Tables

**Table 1:** Primer information of *LhSorPALs* gene.

Gene	Sequence of Forward Primer 5′-3′	The Sequence of Reverse Primer 5′-3′
*qLhSor* *PAL1*	GCCACTGAAGCATTCCGTCTAGC	GCAAGGACAGCGAGGATGTTAGC
*qLhSor* *PAL2*	CCTGTCACCAACCACGTTCAGAG	CACTGCCTCCGCTGTCTTCCTA
*qLhSor* *PAL3*	TCGGGAAGCTCATGTTTGCT	CTGCCCCCTTGAAGCCATAA
*qLhSor* *PAL4*	CAAGGGAGCTGAGATTGCCA	CTCGACGAGATCAACCCGAG
*qLhSor* *PAL5*	TGACTCAAACATCCTCGCCC	TGGCCAGGGTGATGCTTAAG
*qLhSor* *PAL6*	GTGTATGAGGAGGCGATGGG	ACCCCCTACCACTAGTCGAC
*qLhSor* *PAL7*	CAAGGGAGCTGAGATTGCCA	GGTTTGAACTTGGACTCGGTT
*qLhSor* *PAL8*	CTCGCCGGAAGCTCATACAT	CGACGAGATCAACCCGAGAG
*qLhSorEF* (reference gene)	GTTGTGGCTGTGGAGGAAGAAGAG	GACGCAGAACCAAAGAGAGTATCCC

**Table 2:** Primers for prokaryotic expression vector construction.

Name	Sequence (5′-3′)
*LhSorPAL1*-F	ATGGCATCAAAGGACAGCTCC
*LhSorRAL1*-R	GTCTCTGGATTCTTGTATGCTG
*LhSorPAL2*-F	CCTCCTACTCCTCTCCTTCAAC
*LhSorRAL2*-R	CAGCTTAATATAATCACGGTGGG
*LhSorPAL1-BamHI*-F	aaggccatggctgatatcGGATCCatggcacacattgtctgc
*LhSorPAL1-HindIII*-R	gtgctcgagtgcggccgcAAGCTTtcaacaaatcgggagcggc
*LhSorPAL2-BamHI*-F	aaggccatggctgatatcGGATCCatgggacatgtcaacggt
*LhSorPAL2-HindIII-*R	gtgctcgagtgcggccgcAAGCTTttagctgatgggaagggga

**Table 3:** Primers for plant expression vector construction.

Name	Sequence (5′-3′)
OE*-LhSorPAL1-BamHI-*F	tgtttggtgttacttGGATCCatggcacacattgtctgc
OE*-LhSorPAL1-BamHI-*R	caccatgagctcgatGGATCCacaaatcgggagcggc
OE*-LhSorPAL2-BamHI-*F	tgtttggtgttacttggatccGGATCCatgggacatgtcaacggt
OE*-LhSorPAL2-BamHI-*R	caccatgagctcgatGGATCCgctgatgggaagggga
AS*-LhSorPAL1-SacI-*F	tgctcaccatgagctcAATGGGAGTCAATGGGGAGCTG
AS*-LhSorPAL1-BamHI-*R	gtttggtgttacttggatccTCAACAAATCGGGAGCGGCGCG
AS*-LhSorPAL2-SacI-*F	tgctcaccatgagctcGTTGCGAACACGGTAAAGCAGG
AS*-LhSorPAL2-BamHI-*R	gtttggtgttacttggatccTTAGCTGATGGGAAGGGGAGCA

**Table 4:** Formulation of the medium.

Culture Medium Name	Culture Medium Formulation
Infection Suspension	MS + 1.0 mg/L PIC + 10 mmol/L MES + 100 μmol/L AS
Co-culture medium	MS + 1.0 mg/L PIC + 30 g/L sucrose + 10 mmol/L MES + 100 μmol/L AS
screening medium I	MS + 1.0 mg/L PIC + 500 mg/L Cb + 30 g/L sucrose + 9 g/L agar
screening medium II	MS + 1.0 mg/L PIC + 500 mg/L Cb + 2.5 mg/L Basta + 30 g/L sucrose + 9 g/L agar
screening medium III	MS + 2.0 mg/L 6-BA + 0.2 mg/L NAA + 2.5 mg/L Basta + 30 g/L sucrose + 9 g/L agar
rooting medium	MS + 0.01 mg/L TDZ + 0.2 mg/L NAA + 30 g/L sucrose + 9 g/L agar

Abbreviations: PIC, picloram; MES, 2-(N-morpholino)ethanesulfonic acid; AS, acetosyringone; Cb, carbenicillin; Basta, glufosinate-ammonium; 6-BA, 6-benzylaminopurine; NAA, 1-naphthaleneacetic acid; TDZ, thidiazuron.

**Table 5:** Primers for promoter cloning.

Name	Sequence (5′-3′)
*LhSorPAL1*-SP1	CTCCTCCGACAGCTCGACCTTCACC
*LhSorPAL1*-SP2	GGTGGTGCGATTAGAGGGTGCAAGTCTT
*LhSorPAL1*-SP3	CCATCTGTCTAACTTCATCAAGATGGCTTCC
*LhSorPAL1*-SP4	CGTGGGTGAACCGTTTAGGTGAGA
*LhSorPAL1*-SP5	GCGGTGGTGGCTCCACTACATT
*LhSorPAL1*-F	GATGAAGTTAGACAGATGGCAGCAGAA
*LhSorPAL1*-B	AAGCTCAGAACATTTAAAAACA
*LhSorPAL2*-SP1	TGCTTGGTCCTCCGGTGAGAAGTG
*LhSorPAL2*-SP2	CGGTCCCCATGCTCATGCTGTTAATC
*LhSorPAL2*-SP3	AATCACCCACTCACTGCTCGCCTTCAC
*LhSorPAL2*-SP4	AGGTTCTTGTTCGTAATCCTGCTTGA
*LhSorPAL2*-SP5	CGCGGTCAGGACGAGCATAGC
*LhSorPAL2*-B	TGGAACCGATTTGAAGAA

## Data Availability

All data supporting the findings of this study are included within the article and its Supplementary Materials. Additional datasets or raw data are available from the corresponding author upon reasonable request.

## Supplementary Materials

The following supporting information can be downloaded at: https://www.mdpi.com/article/10.3390/plants15121797/s1.

## Abbreviations

The following abbreviations are used in this manuscript:

*Agrobacterium*	*Agrobacterium tumefaciens*
ABA	Abscisic acid
AI	After *B. elliptica* inoculation treatment
AS	Antisense expression strain
*B. cinerea*	*Botrytis cinerea*
*B. elliptica*	*Botrytis elliptica*
Basta	Glufosinate-ammonium
BI	Before *B. elliptica* inoculation treatment
Cb	Carbenicillin
Cef	Cefotaxime
CK	Mock-treated control
*E.coli*	*Escherichia coli*
FPKM	Fragments per kilobase of transcript per million mapped reads
HPLC	High-performance liquid chromatography
hpi	Hours post-inoculation
IPTG	Isopropyl-β-D-thiogalactopyranoside
MeJA	Methyl jasmonate
MES	2-(N-morpholino)ethanesulfonic acid
MS	Murashige and Skoog basal medium
MW	molecular weight
NAA	1-Naphthaleneacetic acid
OE	Overexpression strain
ORF	Open reading frame
PAL	Phenylalanine ammonia-lyase
PDA	Potato dextrose agar
PI	Isoelectric point
PIC	Picloram
SA	Salicylic acid
TDZ	Thidiazuron
WT	Wild type
6-BA	6-Benzylaminopurine
