# Supplementary material for: Botrytis elliptica Infection Induces LhSorPALs Expression in Lilium: Overexpression of LhSorPAL1 and LhSorPAL2 Enhances Disease Resistance via Phenylpropane Metabolite Accumulation
Source: Plants (Basel). 2026 Jun 11;15(12):1797. doi: 10.3390/plants15121797 (PMC13306670; doi:10.3390/plants15121797)
Supplement: Supplementary file 1 [file plants-15-01797-s001.zip › plants-4335878-supplementary.pdf]

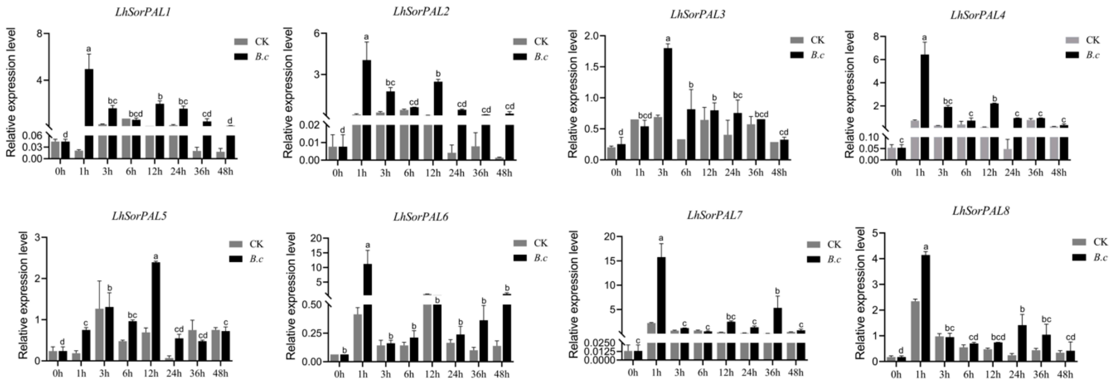

Figure S1. Time course of the level of the *LhSorPAL* genes in ‘Sorbonne’ after infection with *B. cinerea*. Expression levels of eight *LhSorPAL* genes were measured by qRT-PCR at 0, 1, 3, 6, 12, 24, 36, and 48 hpi. Each panel represents one gene. Data are presented as mean ± SD (n = 3). Different lowercase letters above the bars indicate significant differences among time points for each gene ($p < 0.05$).

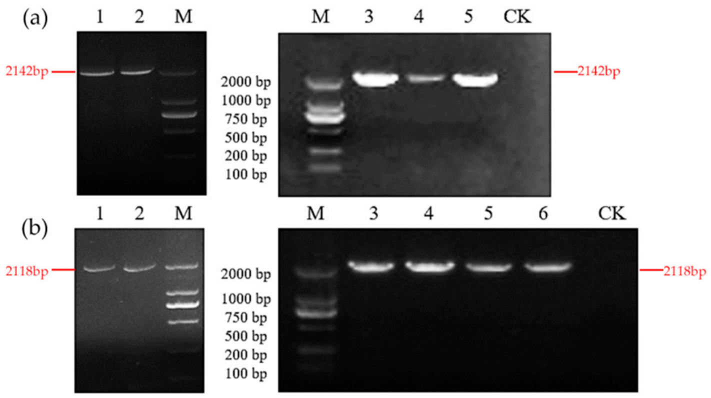

Figure S2. Cloning of *LhSorPAL1* and *LhSorPAL2*. (a) Left: Electrophoretogram of LhSorPAL1 gene clones; right: Electrophoretogram for identification of LhSorPAL1 positive colonies.; (b) Left: Electrophoretogram of *LhSorPAL2* gene clones; right: Electrophoretogram for identification of *LhSorPAL2* positive colonies.; M, DL2000 Marker; CK, Negative control.

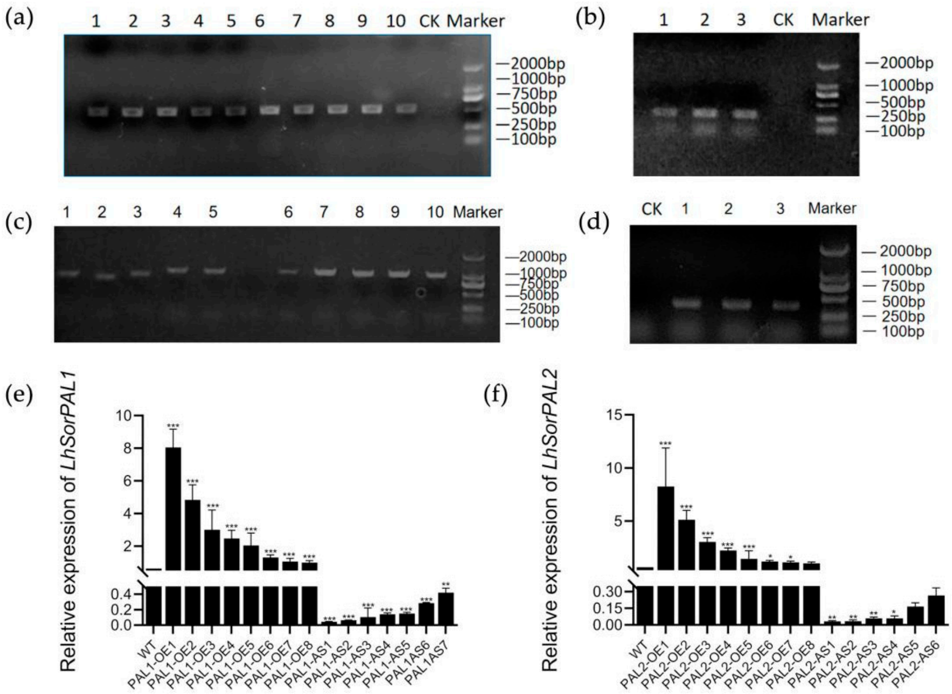

Figure S3. Identification of Genetically Modified Lilies. (a) PCR analysis of *LhSorPAL1* overexpression lines. (b) PCR analysis of *LhSorPAL2* overexpression lines. (c) PCR analysis of *LhSorPAL1* antisense lines. (d) PCR analysis of *LhSorPAL2* antisense lines. (e) *LhSorPAL1* expression levels in transgenic lines. (f) *LhSorPAL2* expression levels in transgenic lines. The plants used in Figures (e) and (f) were derived from the transgenic lines verified by PCR in Figures (a) (b) (c) and (d). Each bar represents an individual plantlet. Due to limitations in the original records, the exact lineage of each plantlet could not be traced.

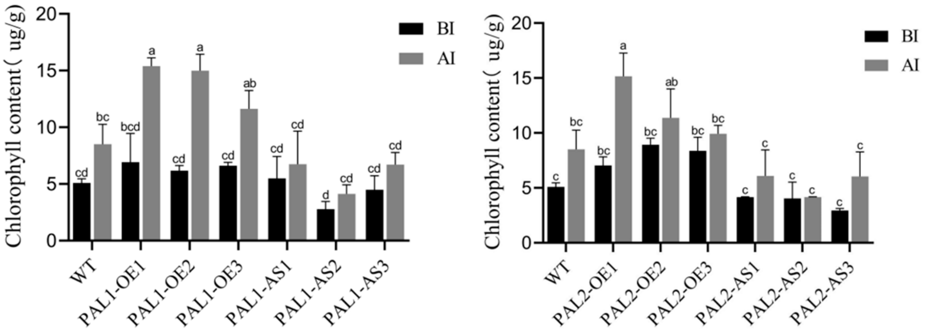

Figure S4. Chlorophyll content of *LhSorPAL1* , *LhSorPAL2* overexpression and antisense plants. WT: wild type; OE: overexpression strain; AS: antisense expression strain.BI: before *B. elliptica* inoculation treatment; AI: after *B. elliptica* inoculation treatment. Three biological replicates were performed for each treatment.

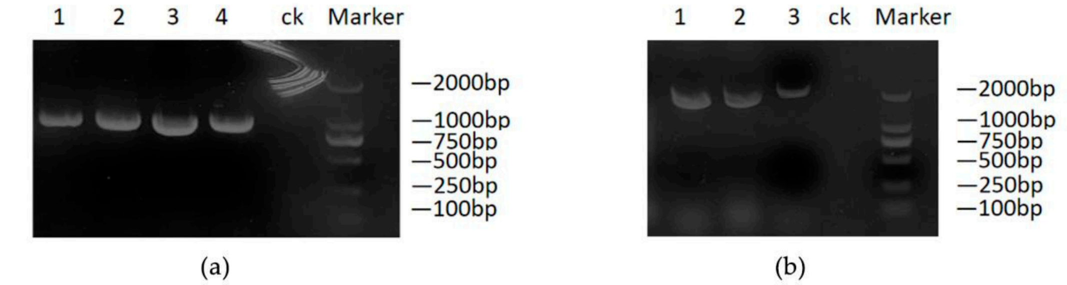

Figure S5. Cloning PCR of *LhSorPAL1* and *LhSorPAL2* promoter. (a) *LhSorPAL1* promoter clone. (b) *LhSorPAL2* promoter clone

Table S1. Analysis of cis-acting elements of the *LhSorPAL1-pro* promoter sequence

Cis-acting element types	Amount	Sequence(5'-3')	Biological function
DRE core	2	GCCGAC	specifically binds to the DREB transcription factor in the induction of adversity (drought, high salinity, low temperature) gene expression
GC-motif	2	CCCCCG	Cis-acting elements involved in the specific induction of hypoxia
LTR	1	CCGAAA	Cis-acting elements involved in the cryogenic response
CAAT-box	12	CAAT/CAAAT/CCAAT	Co-acting elements of promoter and enhancer regions
TATA-box	2	TATA	Core promoter elements around transcription start point -30
MYB	6	CAACCA/CAACAG	Functionality unknown
ABRE	1	ACGTG	Abscisic acid reaction element
TGACG-motif	1	TGACG	Cis-acting regulatory elements involved in the MeJA reaction

Table S2. Analysis of cis-acting elements of the *LhSorPAL2-pro* promoter sequence

Cis-acting element types	Amount	Sequence(5'-3')	Biological function
GC-motif	1	CCCCCG	Cis-acting elements involved in the specific induction of hypoxia
TATA-box	7	TATA/ATATAT/TATATA	Core promoter elements around transcription start point -30
ABRE	2	ACGTG/TACGTGTC	Abscisic acid reaction element
ARE	1	AAACCA	Cis-acting regulatory elements are essential for anaerobic induction
W box	1	TTGACC	It can bind specifically to WRKY transcription factors and plays an important role in inducing gene expression in adverse conditions (drought, high salt, low temperature)
TGACG-motif	4	TGACG	Cis-acting regulatory elements involved in the MeJA reaction
CAAT-box	16	CAAT/CAAAT/CCAAT	Co-acting elements of promoter and enhancer regions
MYC	2	CAATTG/TCTCTTA/CATTTG	Functionality unknown
STRE	3	AGGGG	stress element
GT1-motif	1	GGTTAA	Part of a light-responsive element
TCT-motif	3	TCTTAC	Part of a light-responsive element
Box 4	2	ATTAAT	Cis-acting elements involved in light reactions
MYB	3	CAACTG/TAACCA/CAACCA	Relationship between MYB binding sites and drought inducibility
